# Cellular and Molecular Inflammatory Profile of the Choroid Plexus in Depression and Suicide

**DOI:** 10.3389/fpsyt.2015.00138

**Published:** 2015-10-19

**Authors:** Julia Devorak, Susana Gabriela Torres-Platas, Maria Antonietta Davoli, Josée Prud’homme, Gustavo Turecki, Naguib Mechawar

**Affiliations:** ^1^McGill Group for Suicide Studies, Douglas Mental Health University Institute, Verdun, QC, Canada; ^2^Integrated Program in Neuroscience, McGill University, Montréal, QC, Canada; ^3^Department of Psychiatry, McGill University, Montréal, QC, Canada

**Keywords:** depression, suicide, cytokines, macrophages, choroid plexus, neuroinflammation, human

## Abstract

The inflammatory hypothesis of depression is one of the main theories that endeavors to explain and describe the underlying biological mechanisms of depression and suicide. While mounting evidence indicates altered peripheral and central inflammatory profiles in depressed patients and suicide completers, little is known about how peripheral and central inflammation might be linked in these contexts. The choroid plexus (ChP), a highly vascularized tissue that produces cerebrospinal fluid (CSF) and lacks a blood–brain–barrier, is an interface between peripheral and central immune responses. In the present study, we investigated the cellular and molecular inflammatory profile of the ChP of the lateral ventricle in depressed suicides and psychiatrically healthy controls. Gene expression of macrophages, pro- and anti-inflammatory cytokines, and various factors implicated in immune cell trafficking were measured; and density of ionized calcium-binding adaptor molecule 1-positive (Iba1+) macrophages associated with the ChP epithelial cell layer (ECL) was examined. Significant downregulations of the genes encoding interleukin 1ß (*IL1ß*), a pro-inflammatory acute-phase protein; intercellular cell adhesion molecule 1 (*ICAM1*), a protein implicated in immune cell trafficking in the ChP; and *IBA1*, a monocyte/macrophage marker; were detected in depressed suicides as compared to controls. No difference in the density of Iba1+ macrophages associated with the ChP ECL was observed. While interpretation of these findings is challenging in the absence of corroborating data from the CSF, peripheral blood, or brain parenchyma of the present cohort, we hypothesize that the present findings reflect a ChP compensatory mechanism that attenuates the detrimental effects of chronically altered pro-inflammatory signaling caused by elevated levels of pro-inflammatory cytokines, such as IL-1ß, peripherally and/or centrally. Together, these findings further implicate neuroimmune processes in the etiology of depression and suicide.

## Introduction

Depression affects over 350 million people globally and is the leading cause of disability worldwide (1). Studies have consistently implicated mood disorders, including major depressive disorder (MDD), as a common factor underlying suicide. Approximately 50% of children, adolescents, and adults who die by suicide have a prior mood disorder diagnosis, most commonly MDD (2, 3). Moreover, as many as 15% of individuals with a lifetime diagnosis of MDD admit to having attempted suicide at least once during their lives (4). As the relative contribution of depression to the global burden of disease increases, it becomes increasing important to understand its biological underpinnings, as well as those of suicide, a global leading cause of death that claims the lives of more than 800,000 people annually (5).

The inflammatory hypothesis of depression is one of the main theories that endeavors to explain and describe the underlying biological mechanisms of depression and suicide outcomes. Originally termed the “Macrophage Theory of Depression,” (6) this hypothesis was first formulated based on observations of depressive symptoms precipitated by cytokine therapy in psychiatrically healthy, physically ill human patients [e.g., Ref. (7, 8)]. Maes et al. (9) later expanded this theory in the form of the “Monocyte-T-Lymphocyte Hypothesis of Depression.” A substantial number of studies have been undertaken to investigate the various aspects of this hypothesis, and a large literature has emerged in support of it.

Numerous animal studies have demonstrated that systemic or central administration of pro-inflammatory cytokines induces “sickness behavior,” a spectrum of behavioral signs of sickness, which overlap in large part with the behavioral symptoms of depressive-like states in animals (10, 11). In humans, suicide attempts and completions precipitated by cytokine therapy in psychiatrically healthy, physically ill patients have been reported (12). In addition, human populations suffering from chronic inflammatory conditions, such as type II diabetes, rheumatoid arthritis, and cardiovascular disease, have been reported to show increased incidences of depressive disorders (13). Furthermore, studies measuring peripheral expression of cytokines in depressed human patients have indicated that levels of pro-inflammatory cytokines, such as tumor necrosis factor alpha (TNF-α), interleukin 1 beta (IL-1ß), and interleukin 6 (IL-6), are significantly higher in this clinical population compared to controls [(14–16); for meta-analyses, see Ref. (17–19)]. Interestingly, one study has further indicated that depressed patients who attempt suicide, more specifically, present elevated peripheral levels of pro-inflammatory cytokines TNF-α and IL-6 (20).

Comparatively few studies have investigated inflammatory phenomena in the brain within the context of depression and suicide. Independent groups have reported evidence of increased pro-inflammatory cytokine expression in cortical regions implicated in depression and suicide (21, 22). Furthermore, cellular evidence of increased immune activity in these brain areas has also been provided by our group and others (23–26). How peripheral and central inflammatory phenomena are linked in depression and suicide is a question that requires further investigation.

Several groups have investigated the relationship between cerebrospinal fluid (CSF) and peripheral (blood) cytokine expression in depressed patients and/or suicide attempters [e.g., Ref. (27, 28)]. While these studies provide divergent results with respect to CSF pro-inflammatory cytokine expression in patients versus control subjects, they all report an absence of correlation between CSF and peripheral cytokine expression.

Interestingly, recent evidence from animal models suggests that there is significant trafficking and recruitment of peripherally derived monocytes to the brain under conditions of psychological stress; conditions known to promote anxiety- and depressive-like behaviors. Upon arrival to the brain, these trafficked monocytes differentiate into macrophages that promote inflammatory signaling and influence behavior (29). Recent work by our group (26) and others (25) suggests that a similar monocyte trafficking phenomenon might also occur in the brains of depressed patients. However, in remains to be determined how these purported monocyte–macrophages would gain access to the brain parenchyma.

The choroid plexus (ChP) is a highly vascularized brain structure located along the lateral, third, and fourth ventricles of the brain. It is composed of a continuous, single layer of cuboidal epithelial cells linked by tight junctions, which rests upon a basal lamina and delineates a connective tissue stroma containing a dense bed of fenestrated capillaries (30, 31). It is devoid of most cell types typically found in the brain parenchyma, including astrocytes and microglia (32). While it receives adrenergic, cholinergic, peptidergic, and serotonergic innervations, it does not contain a resident population of neurons. It is, however, home to its own class of resident macrophages, in addition to dendritic cells and other various leukocytes. Best known for the production of CSF, which occurs more precisely at the level of the choroidal epithelium, the ChP also forms part of the blood–CSF barrier (BCSFB) at the level of the epithelial cell tight junctions (30, 31).

Several lines of evidence have implicated the ChP in immune function and in inflammation. It has been demonstrated that the structure is capable of responding to immune modulators, as it constitutively expresses receptors for IL-1ß (i.e., IL1-R1), TNF-α, and IL-6 (33, 34), as well as cell adhesion molecules at the level of the choroidal epithelial cells (35). In addition, several studies have confirmed the ChP’s capacity to produce pro-inflammatory cytokines, such as IL-1ß, TNF-α, and IL-6, as well as chemokines, such as monocyte chemoattractant protein 1 (MCP-1), which has been implicated in leukocyte trafficking (30, 36, 37). Together, these characteristics indicate an important role for the ChP as an interface between peripheral and CNS inflammation.

To our knowledge, few studies have implicated the ChP in depression and suicide. One notable study conducted by Sathyanesan et al. (32) examined gene expression in several brain regions of mice exposed to a chronic unpredictable mild stress, a paradigm known to elicit depressive-like behavioral phenotypes in rodents. This analysis revealed increased expression of pro-inflammatory cytokines TNF-α and IL-1ß in the ChP. More recently, a study conducted by Turner et al. (38) investigated gene expression in postmortem ChP epithelial cell layer of individuals having suffered from MDD. This group reported a downregulation of genes associated with the TGF-ß (transforming growth factor ß) network. These findings might be suggestive of a potential depression-associated inflammatory response. However, it is clear that more work is required to better understand the potential involvement of the ChP in the purported peripheral and central inflammatory responses occurring in depression and suicide.

To this end, the present study aimed to investigate cellular and molecular markers of inflammation in ChP samples from depressed suicides and matched sudden-death controls. Expression of inflammation-associated genes was measured, and the density and distribution of ChP macrophages assessed. The genes investigated included *IL1ß*, *IL6*, and *TNF*α, which encode for pro-inflammatory acute-phase proteins; *IL1R1*, which encodes the receptor of IL-1ß; *IL1RN*, an endogenously occurring antagonist molecule of IL1-R1; *IL10*, an anti-inflammatory cytokine; *MCP1*, a chemokine with monocyte-attracting properties; *ICAM1* (intercellular cell adhesion molecule 1) and *VCAM1* (vascular cell adhesion molecule 1), factors implicated in immune cell trafficking in the ChP; and *IBA1*, a monocyte/macrophage marker (13, 18, 35, 39). In addition, monocyte and/or macrophage trafficking through the ChP epithelium was investigated through quantification of Iba1-IR cells associated with the ChP epithelial cell layer (ECL).

## Materials and Methods

### Samples

This study was conducted with the approval of the Douglas Hospital Research Ethics Board, and with informed consent from next-of-kin. Postmortem brain samples from depressed suicides (DS) and matched, psychiatrically healthy controls (CTRL) were obtained from the Suicide Section of the Douglas-Bell Canada Brain Bank (Douglas Institute). DS subjects died by suicide within the context of a depressive episode, whereas CTRL subjects died suddenly and without history of psychiatric or neurological illness. All DS and CTRL subjects were free of ongoing or prior immune or inflammatory illness. Causes of death were ascertained by the Quebec Coroner’s Office, and psychiatric diagnoses determined by psychological autopsy, as previously described (40). In brief, a trained interviewer conducted the *Structured Clinical Interview for DSM-IV* Psychiatric Disorders (SCID-I) with one or more informants of the deceased. SCID-I assessments, case reports, Coroner’s notes, and medical records were reviewed by a blind panel of clinicians to obtain a consensus diagnosis. Results of toxicological screening were also obtained. Tissue samples were dissected from the choroid plexus of the lateral ventricle. Fresh-frozen samples were used for gene expression analyses (24 cases, 14 controls), and fixed samples for histological analysis (5 cases, 5 controls). Subject groups were matched for age, brain pH, RNA Integrity Number (RIN), and postmortem interval (PMI), defined as the time between death and storage of the body at the morgue (4°C) (Tables 1 and 2).

**Table 1:** **Subject information: gene expression cohort**.

	DS	CTRL
*n*	24	14
Male/female	20/4	11/3
Age (years)	44 ± 2.8	45 ± 5.8
PMI (h)	22.5 ± 3.0	22.5 ± 5.8
Brain pH	6.6 ± 0.1	6.5 ± 0.1
RIN	7.2 ± 0.1	7.3 ± 0.2
Axis 1 disorders	MDD (21); DNOS (3)	Nil (14)
Cause of death	Hanging (16)	Car accident (7)
	Intoxication (5)	Unknown (2)
	Drowning (2)	Medical intoxication (1)
	Jumping (1)	Cardiovascular: cardiac arrest (2)
		Cardiac arrhythmia (1)
		Myocardial infarction (1)

*Groups are statistically similar with respect to age, postmortem interval (PMI), brain pH, and RNA integrity number (RIN). Values are reported as ± SEM*.

*DS, depressed suicide; CTRL, control; MDD, major depressive disorder; DNOS, depressive disorder not otherwise specified*.

**Table 2:** **Subject information: histology cohort**.

	DS	CTRL
*n*	5	5
Male/female	3/2	5/0
Age (years)	45 ± 11.4	50 ± 11.0
PMI (h)	22.9 ± 13.5	13.4 ± 8.3
Brain pH	6.4 ± 0.2	6.8 ± 0.1
Axis 1 disorders	MDD (5)	Nil (5)
Cause of death	Hanging (4)	Car accident (2)
	Intoxication (1)	Other accident (1)
		Cardiac arrest (1)
		Unknown (1)

*Groups are statistically similar with respect to age, postmortem interval (PMI), and brain pH. Values are reported as ± SEM*.

*DS, depressed suicide; CTRL, control; MDD, major depressive disorder; DNOS, depressive disorder not otherwise specified*.

### Gene expression

Total RNA was extracted from 5 to 75 mg of fresh-frozen ChP tissue, depending on availability, using the RNeasy Lipid Tissue Mini Kit (Qiagen Inc., Mississauga, ON, Canada) with an additional DNase digestion performed as per the manufacturer’s instructions. Total RNA content was quantified using a NanoDrop 1000 spectrophotometer (NanoDrop Technologies, Rockland, DE, USA) and RIN determined using an Agilent 2100 Bioanalyzer (Agilent Technologies, Palo Alto, CA, USA). Samples with RIN values below 4.5 were excluded. Total cDNA was synthesized from 1 μg of total RNA using 400 U M-MLV Reverse Transcriptase (Gibco BRL Life Technologies, Burlington, ON, Canada) and oligo-deoxythymidine (dT)-16, as per the manufacturer’s instructions. The following TaqMan Gene Expression Assays, labeled with FAM reporter dye, were used: *IL1ß* (TaqMan Assay ID: Hs01555410_m1), *IL6* (TaqMan Assay ID: Hs99999032_m1), *IL10* (TaqMan Assay ID: Hs99999035_m1), *IL1R1* (TaqMan Assay ID: Hs00168392_m1), *IL1RN* (TaqMan Assay ID: Hs00893626_m1), *AIF1* (henceforth referred to as *IBA1*; TaqMan Assay ID: Hs00610419_g1), *ICAM1* (TaqMan Assay ID: Hs00164932_m1), *VCAM1* (TaqMan Assay ID: Hs01003372_m1), *TNF* (henceforth referred to as *TNF*α; TaqMan Assay ID: Hs00174128_m1), *MCP1* (TaqMan Assay ID: Hs00234140_m1), and *POLR2A* (Taqman Assay ID: Hs00172187_m1). An *ACTB* TaqMan Gene Expression Assay (ID: 4310881E) labeled with VIC reporter dye was also used. Real-time PCR (RT–PCR) reactions were run in quintuplicate with 2 μl of cDNA, 0.6 μl of 20× TaqMan Gene Expression Assay specific to each quantified gene, 6 μl PerfeCTa qPCR Fast Mix (Quanta BioSciences Inc., Gaithersburg, MD, USA) or 6 μl TaqMan Fast Advanced Master Mix (Applied Biosystems, Foster City, CA, USA), and water (milliQ). A standard curve of pooled cDNA from all subjects was used. However, when cytokine expression was too low, a standard curve of cDNA from B-lymphocytes was utilized instead. All samples were analyzed with an ABI PRISM 7900HT Sequence Detection System (Applied Biosytems, Foster City, CA, USA) as per the manufacturer’s instructions using a standard thermal cycling profile. The cycle threshold (CT) values of replicates were pooled to obtain the mean value per subject. Samples with CT standard deviation values above 0.3 were excluded from analysis to avoid excessive variability amongst replicates. Absolute quantification of expression analysis of each gene was performed with housekeeping genes *POLR2A* and *ACTB* as endogenous controls and analyzed using SDS software version 2.4 (Applied Biosystems, Foster City, CA, USA).

### Iba-1 immunohistochemistry

**Figure 1:**
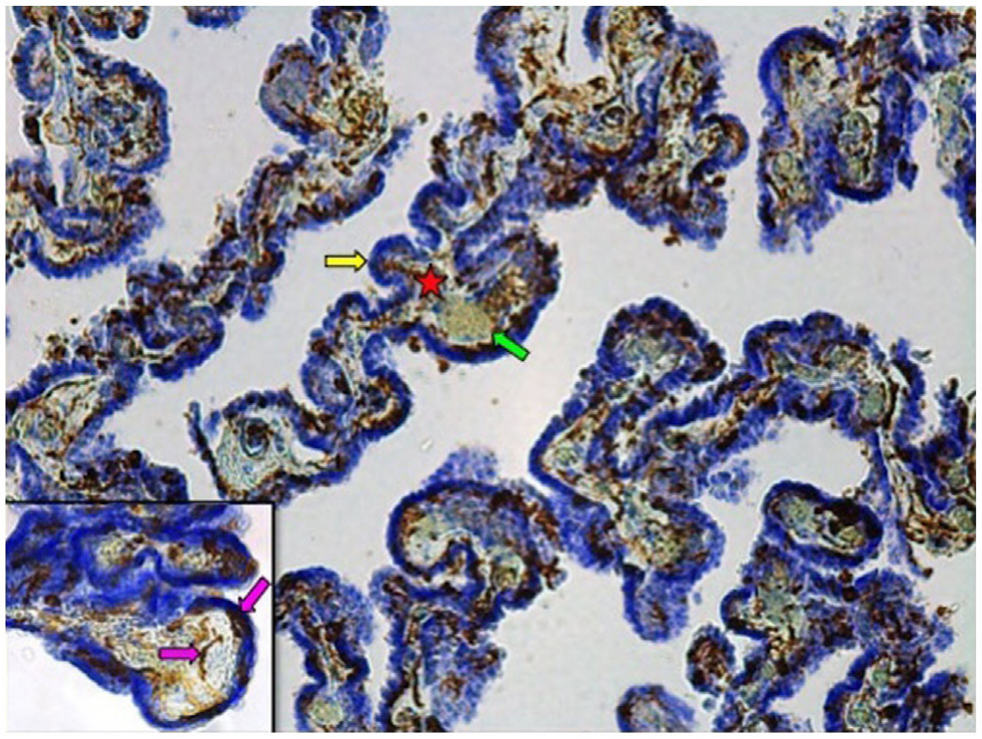
**Iba-1 immunostained choroid plexus with cresyl violet counterstain at 10**×** magnification**. Star denotes connective tissue stroma; yellow arrow indicates epithelial cell layer; green arrow points to blood vessel. Inset: 40× magnification. Pink arrows show Iba1-IR cells associated with the stroma or the epithelial cell layer.

Fixed samples were dehydrated through a graded series of ethanol solutions, cleared in xylene, and embedded in paraffin. Paraffin-embedded tissue was then cut into 40 μm-thick serial sections on a microtome and collected on slides for Iba-1 immunohistochemistry, performed as previously described (26). In brief, tissue sections underwent antigen retrieval by Proteinase K (20 μg/ml) followed by an incubation in 3% H_2_O_2_. Sections were then incubated in a 2% normal goat serum blocking solution overnight before being incubated 24 h in the same blocking solution to which the anti-Iba-1 polyclonal rabbit antibody was added (1:1000; WAKO Chemicals USA, Inc., Richmond, VA, USA). This was followed by a 2 h incubation in blocking solution with a biotinylated goat anti-rabbit secondary antibody (1:1000; Vector Laboratories Inc., Burlington, ON, Canada). Labeling was revealed with a diaminobenzidine kit (Vector Laboratories Inc., Burlington, ON, Canada), and samples were counterstained with cresyl violet to better differentiate ChP compartments (Figure 1).

### Quantitative assessment of epithelial layer-associated Iba1-immunoreactive cells

To estimate ECL-associated Iba1-immunoreactive (-IR) cell densities, a counting area comprising ECL was randomly selected within a subset of Iba1-processed tissue sections with a Zeiss Axio Imager.M2 microscope equipped with a motorized stage and an AxioCam MRc camera. Counting was limited to ECL that was clearly discernible within viewing frames at 10× magnification. Discrete ECL contours were traced using Stereo Investigator software (MBF Bioscience, Williston, VT, USA) and contour areas were calculated using Neurolucida software (MBF Bioscience). 3D virtual tissues encompassing these contours were taken at 40× magnification using Stereo Investigator software. Iba1-IR cells were then quantified using the cell counter plug-in feature of Fiji, an open access software (ImageJ; NIH). Only cells that were located within, or that came into direct contact with, the ECL contours were counted (Figure 1 inset). Given the uneven thickness of the tissue sections following Iba-1 immunohistochemical processing, counting volume was calculated for each discrete contour within a given tissue section, by multiplying the area of each respective contour by the number of *z*-stacks in which the counting was performed. Density was calculated by dividing the total number of Iba1-IR cells by the total volume of counting contours for each subject. These analyses were performed by an investigator (JD) who remained blind to subject groups.

### Statistical analyses

All statistical analyses were performed, and all graphs were produced, using GraphPad Prism 6 (GraphPad Software Inc., La Jolla, CA, USA), unless otherwise noted. All measurements were expressed as mean ± SEM, and a *p* ≤ 0.05 threshold was used to determine statistical significance, unless otherwise noted. *p*-Values exceeding this significance threshold but not exceeding an alpha level of 0.10 were considered statistical trends. Normality was assessed using Shapiro–Wilk tests, and outlier detection was performed using the 3.0IQR method in IBM SPSS Statistics 20 (Statistical Product and Service Solutions, Chicago, IL, USA). For parametric data, two-tailed *t*-tests were used for two-group comparisons, and one-way analyses of variance (ANOVAs) were employed for three-group comparisons. *Post hoc* analyses for parametric 3-group comparisons were conducted using Tukey’s HSD test. When data were non-parametric, two-tailed Mann–Whitney *U* or Kruskal–Wallis tests were used for two-group and three-group comparisons, respectively. *Post hoc* analyses for non-parametric three-group comparisons were conducted using Mann–Whitney *U*-tests with an adjusted alpha level of *p* = 0.0167. Pearson or Spearman correlations were performed on measured variables of normal and non-normal distributions, respectively, to examine the influence of potential confounding factors. When significant correlations were detected, analysis of covariance (ANCOVA) was performed. Non-parametric data sets that positively correlated with confounding factors were transformed using a log_10_ transformation in order to perform ANCOVA.

## Results

### Cytokine, cell adhesion molecule, and chemoattractant gene expression

**Figure 2:**
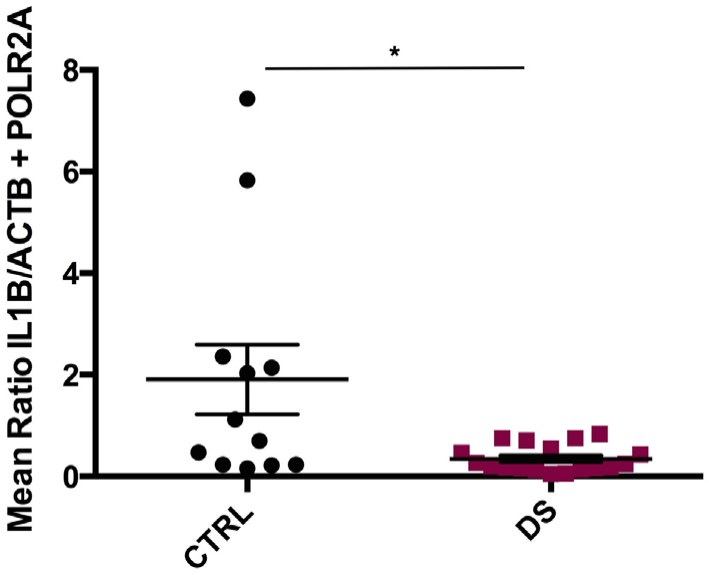
**Absolute expression of *IL1***β** RNA transcript in the choroid plexus of psychiatrically healthy controls (CTRL) and depressed suicides (DS)**. A significant downregulation in *IL1*β expression was observed in DS as compared to CTRL subjects; *p* = 0.013. All values are reported as the mean ratio of *IL1*β expression to the geometric mean expression of endogenous control genes *ACTB* and *POLR2A*.

*IL1ß* mRNA transcript expression was significantly downregulated in DS subjects compared to CTRLs (*U*_(12, 18)_ = 50, *p* = 0.013) (Figure 2). *TNF*α, *IL10*, and *IL6* transcript expressions were also decreased in DS compared to CTRLs; however, these differences did not reach statistical significance. Conversely, *IL1R1* and *IL1RN* gene expression were elevated in DS subjects compared to CTRLs, though these differences did not reach statistical significance either.

**Figure 3:**
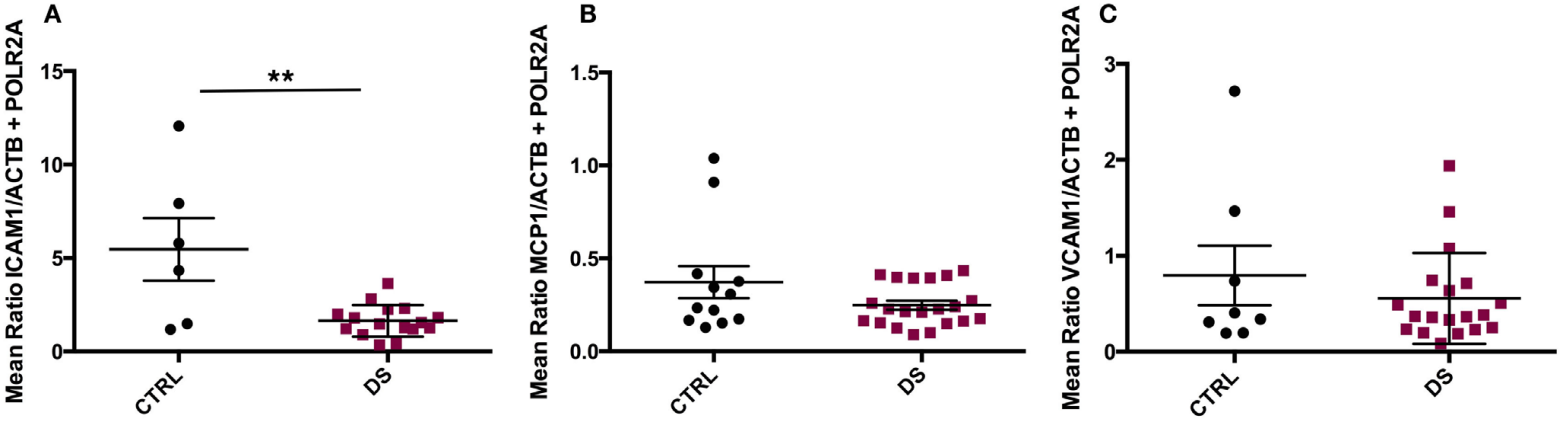
**Absolute RNA transcript expression of blood–CSF barrier-associated molecules in the choroid plexus of psychiatrically healthy controls (CTRL) and depressed suicides (DS)**. **(A)** A significant downregulation in expression of *ICAM1* was observed in DS as compared to CTRL subjects; *p* = 0.002. **(B)** No significant differences in *MCP1* transcript expression were found between groups; *p* > 0.05. **(C)** Average *VCAM1* transcript expression was found to be similar between groups; *p* > 0.05. All values are reported as the mean ratio of expression of probe of interest to the geometric mean expression of endogenous control genes *ACTB* and *POLR2A*.

*ICAM1* gene expression was significantly downregulated in DS compared to CTRL subjects (*t*_(20)_ = 3.647, *p* = 0.002) (Figure 3A), but there were no significant differences in *VCAM1* or *MCP1* transcript expression between groups (Figures 3B,C).

Three-group comparisons were performed to assess the potential effects of antidepressant treatment (ADT) on the expression of the investigated genes, as ADT has been reported to modulate expression levels of peripheral pro-inflammatory cytokines in depressed patients [(41, 42); for a review, see Ref. (43)]. DS subjects were therefore further divided into those receiving ADT around time of death (DS + ADT) and those who were not (DS). ANOVA revealed *ICAM1* to be differentially expressed amongst groups (*F*_(2, 19)_ = 4.376, *p* = 0.027). Subsequent *post hoc* pairwise comparisons revealed that only CTRL and DS differed significantly (*p* = 0.026), with DS expressing lower transcript levels than controls. No significant differences were detected between DS + ADT and DS subjects, or between DS + ADT and CTRL subjects (not shown). Consequently, ADT was not considered a significant confounding factor with respect to *ICAM1* transcript expression.

### IBA1 gene expression and cell density

**Figure 4:**
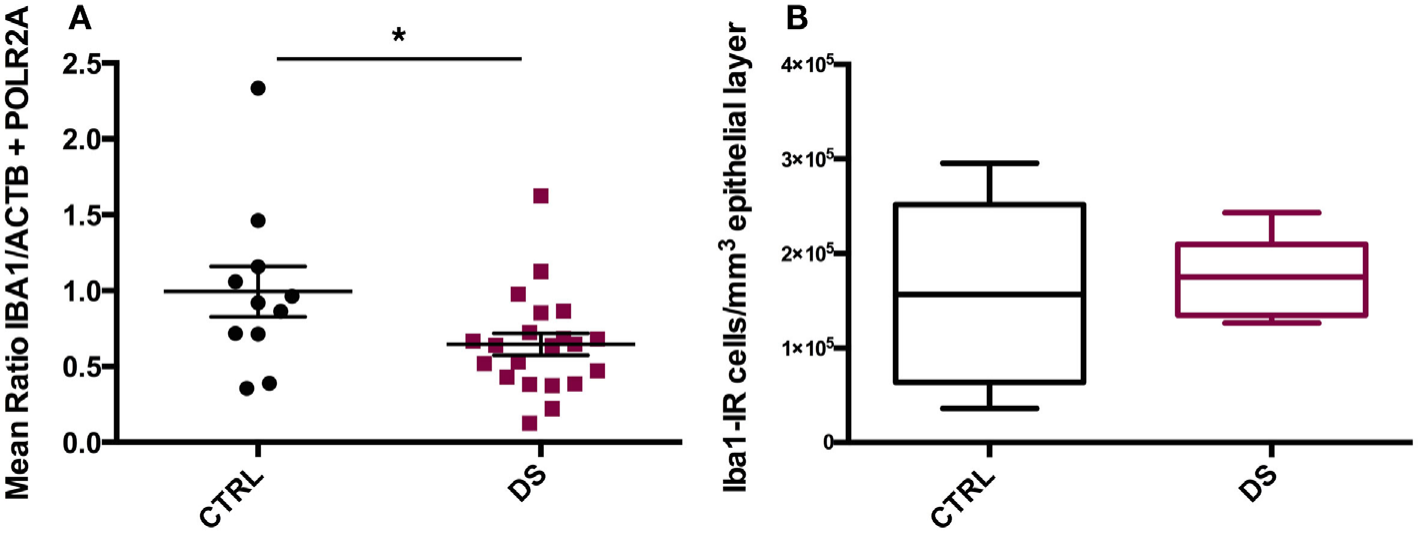
***IBA1* gene expression and immunohistochemical distribution**. **(A)** Absolute expression of *IBA1* RNA transcript in the choroid plexus of psychiatrically healthy controls (CTRL) and depressed suicides (DS). DS expressed significantly less *IBA1* than CTRL subjects; *p* = 0.032. Values are reported as the mean ratio of *IBA1* expression to the geometric mean expression of endogenous control genes *ACTB* and *POLR2A*. **(B)** Quantification of Iba1-IR cells in the choroid plexus epithelial layer of CTRL and DS. No significant difference in epithelial layer Iba1-IR cell number was observed between groups; *p* > 0.10.

*IBA1* mRNA transcript expression was significantly downregulated in DS compared to CTRL subjects (*t*_(20)_ = 2.253, *p* = 0.032) (Figure 4A). However, total densities of ECL-associated Iba1- IR cells did not differ significantly between the two groups (Figure 4B).

### Potential confounding factors

**Figure 5:**
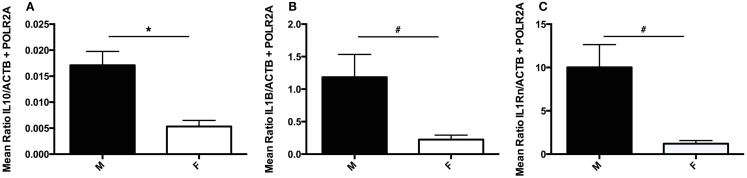
**Sex differences in choroid plexus cytokine gene expression**. **(A)** Absolute expression of *IL10* RNA transcript in male (M) and female (F) subjects. Females expressed significantly less *IL10* than male subjects; *p* = 0.011. **(B)** Absolute expression of *IL1*β RNA transcript in male and female subjects. A trend toward decreased *IL1*β was detected in female versus male subjects, *p* = 0.075. **(C)** Absolute expression of *IL1RN* RNA transcript in male and female subjects. A trend toward decreased *IL1RN* was detected in female versus male subjects, *p* = 0.053. All values are reported as the mean ratio of expression of probe of interest to the geometric mean expression of endogenous control genes *ACTB* and *POLR2A*. #denotes statistical trend, 0.05 < *p* ≤ 0.10.

As males and female have been previously reported to present unique cytokine profiles in the context of suicide (21), potential sex differences were investigated by employing two-group comparisons for each gene investigated. *IL10* transcript expression was found to be significantly downregulated in female subjects as compared to males (*U*_(5, 27)_ = 20, *p* = 0.011) (Figure 5A). As exclusion of female subjects had no impact on *IL10* analyses, female subjects were included in these analyses to increase statistical power. While no additional significant sex-based differences were detected, two statistical trends were revealed: decreased *IL1ß* (*U*_5, 26)_ = 32, *p* = 0.075) and *IL1RN* (*U*_(3, 18)_ = 8, *p* = 0.053) transcript expression in females as compared to males (Figures 5B,C).

The potential confounding effect of substance dependence (SD) on gene expression was also assessed by dividing DS subjects into those with (DS + SD) and without comorbid SD (DS) and then employing three-group comparisons for each gene of interest. ANOVA revealed *IBA1* to be differentially expressed amongst groups (*F*_(2, 28)_ = 3.860, *p* = 0.033). Subsequent *post hoc* pairwise comparisons revealed that only CTRL and DS differed significantly (*p* = 0.031), with DS expressing lower transcript levels than controls. No significant differences were detected between DS + SD and DS subjects, or between DS + SD and CTRL subjects (not shown). Consequently, substance dependence was not considered a significant confounding factor with respect to *IBA1* transcript expression.

Multiple correlations were performed to assess the potentially confounding effects of age, PMI, and tissue pH on gene expression and Iba1-IR cell density. Significant positive correlations between *IL6* mRNA transcript expression and age (ρ = 0.361, *p* = 0.050) and between *IL1R1* mRNA transcript expression and pH (ρ = 0.556, *p* = 0.002) were detected. Analysis of covariance (ANCOVA) revealed no significant difference between DS and CTRL *IL6* expression when controlling for the effect of age, nor any additional significant group comparisons of *IL6* expression after controlling for the effect of age. In addition, ANCOVA revealed no significant difference between DS and CTRL *IL1R1* expression when controlling for the effect of pH, and no further significant group comparisons of *IL1R1* expression after controlling for the effect of pH.

## Discussion

To the best of our knowledge, the present study is the first to specifically investigate inflammatory cells and molecules in the human ChP in the context of depression and suicide. While we found no direct cellular or molecular evidence of inflammation in the ChP, our findings may provide an indirect indication of a central and/or peripheral inflammatory response.

Expression of *IL1ß*, a pro-inflammatory cytokine, was decreased in the ChP samples from depressed suicides. This finding contributes to a heterogeneous body of literature concerning peripheral and central *IL1ß* regulation in human studies of depression and/or suicide. While some postmortem studies have reported both increased mRNA and/or protein expression of this cytokine in prefrontal cortex of depressed patients and/or suicide completers (21, 22), others have reported unaltered expression of this gene in anterior cingulate cortex (26). Studies in clinical populations have suggested elevated levels of IL-1ß in blood (44) and CSF (27) of depressed patients, however unaltered levels of IL-1ß have also been reported in depressed patients and/or suicide attempters (28). Interestingly, the present finding contrasts with that of Sathyanesan et al. (32), who reported increased *IL1ß* gene expression in the lateral ChP of rats exposed to a chronic unpredictable stress paradigm, a well-established animal model of depression.

Interestingly, in addition to *IL1ß* expression, *IBA1* gene expression was also found to be significantly downregulated in depressed suicides. Taken together, these findings could reflect decreased macrophage numbers in the ChP, as ChP macrophages have previously been reported to express IL-1ß (45). However, the present study also revealed unaltered density of ChP ECL-associated Iba1-IR cells, suggesting that any potential decrease in *IBA1* expression would be localized to a ChP compartment other than the ECL.

We can further speculate that the observed decrease in ChP *IL1ß* gene expression in the present study might be indicative of a compensatory mechanism to attenuate the effects of chronically altered central and/or peripheral *IL1ß* expression. One possibility is that altered cerebral *IL1ß* production elicited the proposed compensatory mechanism. Indeed, increased IL-1ß gene and protein expression in the prefrontal cortex (Brodmann areas 8 and 10) of suicides has been reported (22). It is possible that elevated IL-1ß derived from resident brain cells reaches the ChP by way of a reverse nexus pathway described by Johanson et al. (46): after release into the brain interstitial fluid (ISF), IL-1ß would diffuse across a transependymal concentration gradient into the CSF, ultimately reaching the ChP by bulk flow of CSF. To counteract the increased activation of pro-inflammatory signaling pathways in the brain by increased levels of centrally derived IL-1ß, the ChP might function to reduce the amount of CSF-destined IL-1ß it generates, thereby curtailing the total amount of IL-1ß accessing the brain parenchyma. Indeed, ChP epithelial cells are known to produce and secrete cytokines and other intermediate messenger molecules, such as prostaglandins, into the CSF in response to activation by other cytokines (34). Existence of such a phenomenon could help explain reports of unaltered CSF IL-1ß concentrations in depressed patients and suicide attempters [e.g., Ref. (28)]. Alternatively, the ChP might work to attenuate the pro-inflammatory effects that elevated cerebral IL-1ß levels might have on the periphery, via the structure’s extensive network of fenestrated capillaries, by reducing the amount of IL-1ß that is generated by cells associated with the vasculature, such as perivascular macrophages and pericytes. Both of these cell types are known to produce and secrete cytokines in response to their activation by other cytokines (34).

Alternatively, it is possible that the proposed compensatory mechanism arose as a result of altered levels of peripherally derived IL-1ß. Originally reported by Maes et al. (14), elevated plasma and serum IL-1ß protein levels have been reported by several independent groups [for reviews, see Ref. (17–19)]. Cytokines secreted into the bloodstream during infection and systemic inflammation are known to activate cells in the ChP, such as epithelial cells, perivascular macrophages, and pericytes, thereby stimulating production and secretion of cytokines into the CSF. These molecules can then infiltrate the brain ISF and activate resident immune cells (34). In fact, it has been demonstrated that sub-chronic peripheral IL-1ß administration results in increased *IL1ß* mRNA expression in discrete cortical and subcortical areas (47). In the present case, chronically elevated levels of peripherally-derived blood-borne IL-1ß, accessing the ChP by way of the structure’s fenestrated capillaries, might trigger a compensatory decrease in ChP *IL1ß* production. This would curtail the amount of ChP-derived IL-1ß (or other cytokines and intermediate messenger molecules) that could feed back into the bloodstream or gain access to the CSF by an as-of-yet unknown BCSFB transversal mechanism, or both; moreover, it could curtail the ChP’s production of IL-1ß destined for the CSF.

Interestingly, the present findings collectively suggest activation of a compensatory (anti)inflammatory reflex system (CIRS), as has been described by Maes (44) and Maes et al. (48). The CIRS is a constellation of regulatory mechanisms, which attenuate the inflammatory response observed in clinical depression, and which are often accompanied by signs of immunosuppression. These regulatory mechanisms include, but are not limited to, increased production of: interleukin-1 receptor antagonist (IL-1Ra), which inhibits the function of IL-1ß; IL-10, a negative immunoregulatory cytokine; and glucocorticoids and certain acute-phase proteins, which can act as immunosuppressors. While we report no elevation in expression of the genes encoding IL-1Ra (*IL1RN*) or IL-10 at the level of the ChP, we do report gene expression profiles suggestive of CIRS-associated immunosuppression. For example, the observed decrease in *IL1ß* expression and unaltered *IL6* expression might be indicative of the immunosuppressive actions of glucocorticoids, which are known to inhibit production of IL-1ß and IL-6.

Furthermore, it has been hypothesized that the number and function of immune cells, such as monocytes and T cells, might also be negatively regulated by the CIRS (44, 48). IDO activation and reduction of plasma tryptophan levels are thought to be CIRS regulatory mechanisms, and IDO-induced tryptophan reduction and increased tryptophan catabolite (TRYCAT) formation have been shown to attenuate T cell activation and proliferation (48), which could in turn impact monocyte/macrophage number and function. The presently observed decrease in ChP *IBA1* expression might therefore be indicative of a CIRS-associated decrease in number of ChP macrophages. Furthermore, decreased *IBA1* expression in conjunction with decreased *IL1ß* expression might be indicative of a CIRS-associated decline in function of ChP monocytes and/or macrophages.

The present results also indicate a downregulation in ChP *ICAM1* gene expression in depressed suicides. As ICAM-1 is expressed by choroid epithelial cells and is thought to support immune cell trafficking in the ChP (35, 49), this finding might be an indication of attenuated immune cell trafficking through the BCSFB in depression and suicide. ICAM-1 in the choroidal epithelium is also known to be upregulated in response to acute pro-inflammatory molecule exposure *in vitro* (49). It is possible that the presently observed downregulation of *ICAM1* expression in the choroid plexus might represent a compensatory mechanism that functions to counteract (chronically) increased activation of pro-inflammatory signaling pathways elicited by chronically elevated cytokine levels frequently observed in depressed patients. An attenuation of immune cell trafficking into or out of the structure by way of the BCSFB could consequently curtail central and/or peripheral pro-inflammatory signaling. Interestingly, the observed decrease in ChP *IBA1* expression in depressed suicides could reflect such an attenuation of immune cell trafficking into the ChP – more specifically, that of monocytes and/or macrophages. In addition, the observed unaltered density of ChP ECL-associated Iba1-IR cells in DS subjects could further suggest a compensatory attenuation of monocytes and/or macrophages trafficking into the ChP.

Several confounding factors may have influenced the present results, and these accordingly merit discussion. One potential confounding factor in both the gene expression and histological analyses was the heterogeneity of the tissue samples. Most tissues were dissected, with a reasonable degree of certainty, from the choroid plexus of the lateral ventricle. However, tissue was not consistently sampled from the same region of the lateral ventricle ChP, often due to varying tissue availability. While it is unclear whether regional differences exist within the human ChP, by virtue of which inconsistent tissue sampling could affect the present analyses, the possibility cannot be ruled out.

A second potentially confounding factor, for gene expression analyses exclusively, was the heterogeneity of the depressed suicide group. Several independent groups have demonstrated that differing peripheral cytokine profiles exist among MDD subtypes, including melancholic, atypical, and treatment resistant [e.g., see Ref. (50–53)]. Furthermore, different stages of depression have been shown to present unique cytokine expression profiles (51). The present DS cohort contained several subjects who did not have a diagnosis of MDD, but rather a depressive disorder not otherwise specified. In addition, depression subtype and staging were unknown for these subjects, as this retrospective data was not available. Another confounding factor, for gene expression analyses exclusively, was the presence of considerable quantities of blood in the frozen ChP tissue. As it was impossible to remove the blood from the ChP tissue, RNA was extracted, and cDNA was synthesized, from ChP tissue together with the blood it contained. Consequently, the gene expression analyses conducted in this study reflect ChP-derived gene transcripts as well as peripherally-derived transcripts of unknown quantities.

Lastly, in addition to the aforementioned experimental confounds, the present study presents other limitations such as small sample size and lack of retrospective data for both CTRL and DS cohorts. Small sample size was due to the limited number of ChP tissue samples meeting the criteria for our study, in conjunction with the exclusion criteria employed in analyzing replicates for each probe of interest, in the gene expression experiments. Certain retrospective data, such as smoking, BMI, and disease subtype and staging, were often unavailable.

In conclusion, this exploratory study is the first to provide evidence for altered gene expression profiles of pro-inflammatory cytokines, immune cells, and factors implicated in immune cell trafficking, in the choroid plexus of depressed suicides. *IL1ß*, *ICAM1*, and *IBA1* gene expression were all found to be downregulated in depressed suicides. Together, these findings are suggestive of a compensatory mechanism at the level of the ChP that might function to attenuate a chronically activated inflammatory response. While these findings do not provide direct evidence of ChP inflammation in the context of depression and suicide, the altered gene expression profiles described herein may indirectly indicate pro-inflammatory phenomena occurring peripherally and/or centrally. Overall, these findings provide further evidence that neuroimmune processes are implicated in depression and suicide.

## Author Contributions

JD was involved in the design and execution of this study, the optimization and execution of most experiments, and the collection and analysis of most data. GT contributed to the interpretation of the data. NM contributed to and coordinated the design and execution of all aspects of the study, including conception, data interpretation, and analysis. The study was supported by NM, and the manuscript prepared by JD and NM in consultation with SGTP, MAD, and JP.

## Conflict of Interest Statement

The authors declare that the present research was conducted in the absence of any commercial or financial relationships that could be construed as potential conflicts of interest.
